# Global, regional, and national consumption of animal-source foods between 1990 and 2018: findings from the Global Dietary Database

**DOI:** 10.1016/S2542-5196(21)00352-1

**Published:** 2022-03-09

**Authors:** Victoria Miller, Julia Reedy, Frederick Cudhea, Jianyi Zhang, Peilin Shi, Josh Erndt-Marino, Jennifer Coates, Renata Micha, Patrick Webb, Dariush Mozaffarian, Global Dietary Database, Pamela Abbott, Morteza Abdollahi, Parvin Abedi, Suhad Abumweis, Linda Adair, Mohannad Al Nsour, Nasser Al-Daghri, Nawal Al-Hamad, Suad Al-Hooti, Sameer Al-Zenki, Iftikhar Alam, Jemal H Ali, Eman Alissa, Simon Anderson, Karim Anzid, Carukshi Arambepola, Mustafa Arici, Joanne Arsenault, Renzo Asciak, Helene E Barbieri, Noël Barengo, Simon Barquera, Murat Bas, Wulf Becker, Sigrid Beer-Borst, Per Bergman, Lajos Biró, Sesikeran Boindala, Pascal Bovet, Debbie Bradshaw, Noriklil BI Bukhary, Kanitta Bundhamcharoen, Mauricio Caballero, Neville Calleja, Xia Cao, Mario Capanzana, Jan Carmikle, Katia Castetbon, Michelle Castro, Corazon Cerdena, Hsing-Yi Chang, Karen Charlton, Yu Chen, Mei F Chen, Shashi Chiplonkar, Yoonsu Cho, Khun-Aik Chuah, Simona Costanzo, Melanie Cowan, Albertino Damasceno, Saeed Dastgiri, Stefaan De Henauw, Karin DeRidder, Eric Ding, Rivera Dommarco, Rokiah Don, Charmaine Duante, Vesselka Duleva, Samuel Duran Aguero, Veena Ekbote, Jalila El Ati, Asmaa El Hamdouchi, Tatyana El-kour, Alison Eldridge, Ibrahim Elmadfa, Alireza Esteghamati, Zohreh Etemad, Fariza Fadzil, Farshad Farzadfar, Anne Fernandez, Dulitha Fernando, Regina Fisberg, Simon Forsyth, Edna Gamboa-Delgado, Didier Garriguet, Jean-Michel Gaspoz, Dorothy Gauci, Marianne Geleijnse, Brahmam Ginnela, Giuseppe Grosso, Idris Guessous, Martin Gulliford, Ingibjorg Gunnarsdottir, Wilbur Hadden, Aida Hadziomeragic, Christian Haerpfer, Rubina Hakeem, Aminul Haque, Maryam Hashemian, Rajkumar Hemalatha, Sigrun Henjum, Hristo Hinkov, Zaiton Hjdaud, Daniel Hoffman, Beth Hopping, Anahita Houshiar-rad, Yao-Te Hsieh, Shu-Yi Hung, Inge Huybrechts, Nahla C Hwalla, Hajah M Ibrahim, Nayu Ikeda, Daniel Illescas-Zarate, Manami Inoue, Chandrashekar Janakiram, Ranil Jayawardena, Rajesh Jeewon, Nattinee Jitnarin, Lars Johansson, Olof Jonsdottir, Ahvaz Jundishapur, Ola Kally, Mirnalini Kandiah, Tilakavati Karupaiah, Lital Keinan-Boker, Roya Kelishadi, Anuradha Khadilkar, Cho-il Kim, Eda Koksal, Jurgen Konig, Liisa Korkalo, Jeremy Koster, Irina Kovalskys, Anand Krishnan, Herculina Kruger, Rebecca Kuriyan-Raj, Sanghui Kweon, Carl Lachat, Yuen Lai, Pulani Lanerolle, Avula Laxmaiah, Catherine Leclercq, Meei-Shyuan Lee, Hae-Jeung Lee, Eva W Lemming, Yanping Li, Jaana Lindström, Annie Ling, Nur IL Liputo, Patricio Lopez-Jaramillo, Amy Luke, Widjaja Lukito, Elisabette Lupotto, Yi Ma, Zaleha A Mahdy, Reza Malekzadeh, Wan Manan, Dirce Marchioni, Lydia L Marques, Pedro Marques-Vidal, Yves Martin-Prevel,, Angie Mathee, Yasuhiro Matsumura, Paramita Mazumdar, Anjum Memon, Gert Mensink, Alexa Meyer, Parvin Mirmiran, Masoud Mirzaei, Puneet Misra, Anoop Misra, Claudette Mitchell, Hamid JBJ Mohamed, Fatemeh Mohammadi-Nasrabadi, Noushin Mohammadifard, Foong M Moy, Abdulrahman Musaiger, Elizabeth Mwaniki, Jannicke Myhre, Balakrishna Nagalla, Androniki Naska, Swee A Ng, Shu W Ng, Le TN Ngoan, Sina Noshad, Angelica Ochoa, Marga Ocke, Jillian Odenkirk, Kyungwon Oh, Mariana Oleas, Sonia Olivares, Philippos Orfanos, Johana Ortiz-Ulloa, Johanna Otero, Marja-Leena Ovaskainen, Mohammadreza Pakseresht, Cristina Palacios, Pam Palmer, Wen-Harn Pan, Demosthenes Panagiotakos, Rajendra Parajuli, Myungsook Park, Gulden Pekcan, Stefka Petrova, Noppawan Piaseu, Christos Pitsavos, Kalpagam Polasa, Luz Posada, Farhad Pourfarzi, Alan M Preston, Ingrid Rached, Ali R Rahbar, Colin Rehm, Almut Richter, Leanne Riley, Benoit Salanave, Luz M Sánchez-Romero, Nizal Sarrafzadegan, Norie Sawada, Makiko Sekiyama, Rusidah Selamat, Khadijah Shamsuddin, Zalilah M Shariff, Sangita Sharma, Abla M Sibai, Harri Sinkko, Isabelle Sioen, Ivan Sisa, Sheila Skeaff, Laufey Steingrimsdottir, Tor Strand, Milton F Suarez-Ortegon, Sumathi Swaminathan, Gillian Swan, Elzbieta Sygnowska, Maria Szabo, Lucjan Szponar, Ilse Tan-Khouw, Heli Tapanainen, Reema Tayyem, Bemnet Tedla, Alison Tedstone, Robert Templeton, Celine Termote, Anastasia Thanopoulou, Holmfridur Thorgeirsdottir, Inga Thorsdottir, Dimitrios Trichopoulos, Antonia Trichopoulou, Shoichiro Tsugane, Aida Turrini, Coline van Oosterhout, Erkki Vartiainen, J Lennert Veerman, Suvi Virtanen, Peter Vollenweider, Marieke Vossenaar, Indu Waidyatilaka, Anna Waskiewicz, Eveline Waterham, Lothar Wieler, Tizita Wondwossen, Suh Wu, Roseyati Yaakub, Mabel Yap, Safiah Yusof, Sahar Zaghloul, Gábor Zajkás, Maria Zapata, Khairul Zarina, Fatemeh V Zohoori

**Affiliations:** a Friedman School of Nutrition Science and Policy, Tufts University, Boston, MA, USA; b Department of Food Science and Nutrition, University of Thessaly, Volos, Greece

## Abstract

**Background:**

Diet is a major modifiable risk factor for human health and overall consumption patterns affect planetary health. We aimed to quantify global, regional, and national consumption levels of animal-source foods (ASF) to inform intervention, surveillance, and policy priorities.

**Methods:**

Individual-level dietary surveys across 185 countries conducted between 1990 and 2018 were identified, obtained, standardised, and assessed among children and adults, jointly stratified by age, sex, education level, and rural versus urban residence. We included 499 discrete surveys (91·2% nationally or subnationally representative) with data for ASF (unprocessed red meat, processed meat, eggs, seafood, milk, cheese, and yoghurt), comprising 3·8 million individuals from 134 countries representing 95·2% of the world population in 2018. We used Bayesian hierarchical models to account for differences in survey methods and representativeness, time trends, and input data and modelling uncertainty, with five-fold cross-validation.

**Findings:**

In 2018, mean global intake per person of unprocessed red meat was 51 g/day (95% uncertainty interval [UI] 48–54; region-specific range 7–114 g/day); 17 countries (23·9% of the world's population) had mean intakes of at least one serving (100 g) per day. Global mean intake of processed meat was 17 g/day (95% UI 15–21 g/day; region-specific range 3–54 g/day); seafood, 28 g/day (27–30 g/day; 12–44 g/day); eggs, 21 g/day (18–24 g/day; 6–35 g/day); milk 88 g/day (84–93 g/day; 45–185 g/day); cheese, 8 g/day (8–10 g/day; 1–34 g/day); and yoghurt, 20 g/day (17–23 g/day; 7–84 g/day). Mean national intakes were at least one serving per day for processed meat (≥50 g/day) in countries representing 6·9% of the global population; for cheese (≥42 g/day) in 2·3%; for eggs (≥55 g/day) in 0·7%; for milk (≥245 g/day) in 0·3%; for seafood (≥100 g/day) in 0·8%; and for yoghurt (≥245 g/day) in less than 0·1%. Among the 25 most populous countries in 2018, total ASF intake was highest in Russia (5·8 servings per day), Germany (3·8 servings per day), and the UK (3·7 servings per day), and lowest in Tanzania (0·9 servings per day) and India (0·7 servings per day). Global and regional intakes of ASF were generally similar by sex. Compared with children, adults generally consumed more unprocessed red meat, seafood and cheese, and less milk; energy-adjusted intakes of other ASF were more similar. Globally, ASF intakes (servings per week) were higher among more-educated versus less-educated adults, with greatest global differences for milk (0·79), eggs (0·47), unprocessed red meat (0·42), cheese (0·28), seafood (0·28), yoghurt (0·22), and processed meat (0·21). This was also true for urban compared to rural areas, with largest global differences (servings per week) for unprocessed red meat (0·47), milk (0·38), and eggs (0·20). Between 1990 and 2018, global intakes (servings per week) increased for unprocessed red meat (1·20), eggs (1·18), milk (0·63), processed meat (0·50), seafood (0·44), and cheese (0·14).

**Interpretation:**

Our estimates of ASF consumption identify populations with both lower and higher than optimal intakes. These estimates can inform the targeting of intervention, surveillance, and policy priorities relevant to both human and planetary health.

**Funding:**

Bill & Melinda Gates Foundation and American Heart Association.

## Introduction

Diet is a major modifiable risk factor for maternal and child health, undernutrition, and non-communicable diseases (NCDs) worldwide.^1^ The food that humans consume is also a major determinant of environmental sustainability, with impacts on land, freshwater, energy use, greenhouse gas emissions, deforestation, and biodiversity loss.^2^, ^3^ Among the different food groups that influence both human and planetary health, perhaps none is more relevant than animal-source foods (ASF).^4^ ASF are diverse and heterogeneous, including unprocessed red meat, processed meat, poultry, eggs, seafood, milk, cheese, and yoghurt. These foods often contain high and bioavailable contents of important nutrients, for example vitamin A, folic acid, calcium, iodine, iron, zinc, essential fatty acids, and protein,^5^, ^6^, ^7^ are the only natural dietary sources of vitamins B12 and D,^8^, ^9^ and can have higher levels of some nutrients (eg, vitamin A, folate, vitamin B12, calcium, iron, and zinc) than plant-source foods.^10^ These characteristics make ASF useful for improving nutrition in vulnerable populations such as infants, young children, adolescents, women of reproductive age, pregnant and lactating women, and older adults, as well as very poor communities in low-income and middle-income countries.^6^, ^11^, ^12^, ^13^ Different ASF might also have important adverse (eg, processed meats) or beneficial (eg, seafood and yoghurt) effects on NCDs, in particular cardiovascular disease, diabetes, and cancer.^14^, ^15^ Impacts on planetary health are just as important, including increased greenhouse gas emissions, land and energy use, and acidification and eutrophication, and also vary according to the type of ASF,^16^, ^17^, ^18^ production method, and the suitability of the specific ASF to the local ecosystem in which it is produced.^19^, ^20^

**Research in context**

**Evidence before this study**

We systematically searched PubMed, Embase, Web of Science, LILACS, African Index Medicus, and the Southeast Asia Index Medicus to identify studies reporting nationally or subnationally representative estimates of individual-level consumption of seven animal-source foods (ASF). We included 1248 studies conducted between 1980 and 2016 using 24-h recalls, food frequency questionnaires, or short standardised questionnaires. When national or subnational individual-level surveys were not identified for a country, we searched for individual-level surveys from large cohorts, the WHO Global Infobase, and the WHO Stepwise Approach to Surveillance database. Household budget surveys were used on the rare occasion when individual-level dietary surveys were not identified for a populous country. We excluded surveys focused on special populations or cohorts. Using Bayesian hierarchical modelling methods, we estimated global, regional, and national intakes of ASF by age, sex, education, urbanicity, and time between 1990 and 2018.We identified one previous global analysis of ASF consumption, which was limited to a few ASF subtypes, used crude national estimates of food availability or expenditure data to estimate individual-level intakes, and did not report consumption by important socioeconomic factors.

**Added value of this study**

This study provides a comprehensive picture of consumption of total ASF, unprocessed red meat, processed meat, seafood, egg, milk, cheese, and yoghurt consumption in 185 countries among children (aged ≤19 years) and adults (≥20 years). It includes the first global estimates of mean ASF intakes by education level and urbanicity. We also present trends in ASF consumption over three decades. To our knowledge, this is the most current and comprehensive study on global individual-level consumption of ASF.

**Implications of all the available evidence**

This study highlights regions, countries, and population strata with both lower and higher than optimal ASF intakes. The findings can inform the targeting of intervention, surveillance, and policy priorities relevant to both human and planetary health

Based on these important and heterogeneous effects of ASF on both human and planetary health, it is crucial to understand the patterns and distributions of their consumption globally, not only across countries but also within population subgroups such as by age, sex, education level, and urban versus rural residence. However, such distributions around the world are not well established. Previous reports are not up-to-date,^21^, ^22^ reported on only a few ASF subtypes,^23^ used crude national estimates of food availability or expenditure^23^ that may not reflect dietary intakes,^24^ or are focused on select countries, regions, or age groups.^25^, ^26^, ^27^, ^28^, ^29^ Furthermore, global consumption levels among children or by education attainment and urban versus rural residence have not been previously reported.

To address these key knowledge gaps and provide the best current evidence on dietary intakes of key ASF worldwide, we aimed to systematically collect and evaluate global data from national and subnational individual-level dietary surveys using comparable and standardised methods and developed and applied Bayesian hierarchical modelling methods to estimate consumption of ASF by world region, country, age, sex, education level, and urbanicity, alongside changes in time between 1990 and 2018.

## Methods

### Data sources

The Global Dietary Database (GDD) is an international collaborative effort to produce comprehensive and comparable estimates of dietary intakes of major foods and nutrients in 185 countries and territories. Details on methods and the standardised data collection protocol have been described.^21^, ^22^, ^30^, ^31^, ^32^, ^33^, ^34^ Briefly, we performed systematic searches for individual-level dietary surveys in countries and territories globally, as well as extensive personal communications with researchers and government authorities throughout the world, inviting them to be corresponding members of the GDD. The results of our search strategy by dietary factor, time, and region have been detailed.^30^ Surveys were prioritised if nationally or subnationally representative and using individual-level dietary assessments with standardised 24-h recalls, food frequency questionnaires, or short standardised questionnaires (eg, Demographic Health Survey questionnaires). When national or subnational individual-level surveys were not identified for a country, we searched for individual-level surveys from large cohorts, the WHO Global Infobase, and the WHO Stepwise Approach to Surveillance database. Household budget surveys were used on the rare occasion when individual-level dietary surveys were not identified for a populous country. We excluded surveys focused on special populations (eg, pregnant or nursing women, or individuals with a specific disease) or cohorts (eg, a specific occupation or dietary pattern).

The final GDD model incorporated 1248 dietary surveys representing 188 countries and 99·0% of the global population in 2018. Of these surveys, 499 (40%) reported data for ASF including unprocessed red meat, total processed meat (ie, any meat, including poultry, that was cured, smoked, dried, or chemically preserved, excluding seafood and eggs), seafood, egg, milk, cheese, and yoghurt consumption (appendix pp 9–11). Based on the original focus on factors with potential causal associations with NCDs, the data collection did not include poultry, which we hope to update in future iterations. The 499 surveys included 3·8 million individuals from 134 countries, representing 95·2% of the global population. Most surveys were nationally or subnationally representative (91·2%), collected at the individual-level (80·7%), and included data for children and adolescents (93·4%), adults (76·3%), by rural or urban residence status (66·5%), and by education level (50·2%).

### Data extraction

For each survey, we extracted data using standardised methods on survey characteristics and dietary metrics, units, mean, and standard deviation of consumption, by age, sex, education level, and urban or rural residence.^31^ Data were assessed for extraction errors and for plausibility using standardised algorithms, and survey quality by evaluating evidence for selection bias, sample representativeness, response rate, and validity of diet assessment method (appendix pp 3–5). Measurement comparability across surveys was maximised by using a standardised data analysis approach that leveraged the average of all days of dietary assessment to quantify mean individual-level intakes; used harmonised dietary factor definitions and units of measure across surveys; and adjusted for total energy using age-specific energy intakes to reduce measurement error and account for differences in body size, metabolic efficiency, and physical activity (appendix pp 3–8). All intakes are reported adjusted to 700 kcal/day for ages <1 year, 1000 kcal/day for ages 1–<2 years, 1300 kcal/day for ages 2–5 years, 1700 kcal/day for ages 6–10 years, 2000 kcal/day for ages 11–74 years, and 1700 kcal/day for ages ≥75 years.

### Data modelling

To incorporate and address differences in data comparability, and sampling uncertainty, we used a Bayesian model with a nested hierarchical structure (with random effects by country, region, and globally) to estimate the mean consumption level of each ASF and its statistical uncertainty for each of the 264 population strata across 185 countries and each year between 1990 and 2018. Stratifying factors included age (<1, 1–2, 3–4, 5–9, 10–14, 15–19, 20–24, 25–29, 30–34, 35–39, 40–44, 45–49, 50–54, 55–59, 60–64, 65–69, 70–74, 75–79, 80–84, 85–89, 90–94, and ≥95 years), sex, education (≤6 years of education, >6 years to <12 years, or ≥12 years), and urbanicity (urban or rural), jointly stratified. For each dietary factor, primary inputs were the survey-level quantitative data (by country, time, age, sex, education level, and urban or rural-status); survey characteristics (dietary assessment method, type of dietary metric); and country-year-specific covariates (appendix pp 12–13). The model included overdispersion of study-level variance for surveys that were not nationally representative or not stratified by smaller age groups (≤10 years), sex, education level, or urbanicity. Uncertainty of each stratum-specific estimate was quantified using 4000 iterations to determine posterior distributions of consumption jointly by country, year, and demographic subgroup. We computed the median intake and the 95% uncertainty interval (UI) for each stratum as the 50th, 2·5th and 97·5th percentiles of the 4000 draws, respectively. Validity was assessed by five-fold cross-validation (randomly omitting 20% of the raw survey data, run five times), comparing predicted versus observed intakes; as well by assessment of implausible estimates and visual assessment of global and national mean intakes using heat maps. A second time component Bayesian model was used to strengthen time trend estimates for dietary factors with corresponding food or nutrient availability data (FAO Food Balance Sheet^35^ and Global Expanded Nutrient Supply^36^). The model incorporated country-level intercepts and slopes, along with their correlation that is estimated across countries. This model is commonly referred to as a varying slopes model structure and leverages two-dimensional partial pooling between intercepts and slopes to regularise all parameters and minimise overfitting risk.^37^, ^38^ The final presented results are a combination of these two separate Bayesian models, as specified in detail in appendix pp 14–22.

### Statistical analysis

Global, regional, national, and within-country population subgroup intakes and their uncertainty were calculated as population-weighted averages using all 4000 posterior predictions for each of the 264 demographic strata in each country-year. Population weights for each year were derived from the United Nations Population Division,^39^ supplemented with data for education and urban versus rural status from Barro and Lee^40^ and the United Nations.^41^ Intakes were calculated as both grams per day (g/day) and servings per day or week, using standardised portion sizes. Spearman correlations between national mean intakes of different ASF were assessed. Changes in consumption between 1990 and 2018 were calculated at the stratum-specific prediction level to account for the full spectrum of uncertainty and standardised to the proportion of individuals within each stratum in 2018 to account for changes in demographics over time. Absolute and percentage differences in consumption between population subgroups were similarly calculated, using all 4000 posterior predictions for each stratum and population-weights in 2018. Differences in education level were computed as the difference for high (≥12 years) versus low (<6 years) education excluding moderate education (between 6 years and <12 years). For comparisons between sexes, education levels, urbanicity, and time, difference thresholds were regarded as significant if the 95% UI did not include zero. Given the Bayesian estimates, no formal alpha level should be defined for statistical significance; and 95% UIs of each estimate should be considered as a guide.

### Role of the funding source

The Bill & Melinda Gates Foundation contributed to study design during the grant application process; the funders otherwise had no role in data collection, data analysis, data interpretation, or writing of the report.

## Results

**Figure 1:**
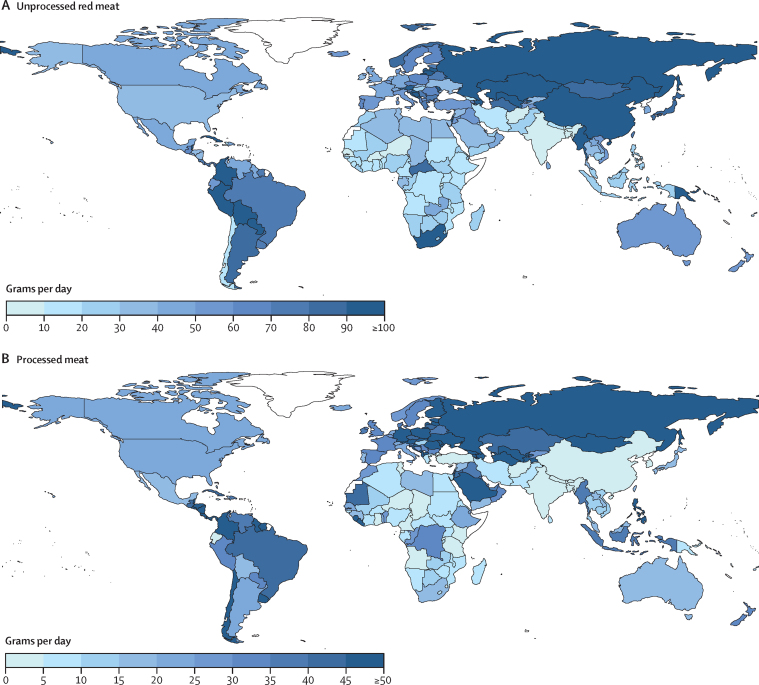
National mean intake of unprocessed red meat (A) and processed meat (B) in 2018, all ages 17 (9%) of 185 countries consumed at least 100 g/day (equivalent to one serving per day) of unprocessed red meat, representing 1·8 billion people or 23·9% of the world population; 32 (17%) of 185 countries had intakes of at least 50 g/day (equivalent to one serving per day) of processed meat, representing 520 million people or 6·9% of the world population.

In 2018, mean global consumption of unprocessed red meat was 51 g/day (95% UI 48–54), with a 16-fold variation across seven geographical regions (from 7 g/day in South Asia to 114 g/day in Central or Eastern Europe and Central Asia; table). Among the world's 25 most populous countries, mean national intakes were highest in Russia, South Africa, China, and Japan, and lowest in India, Bangladesh, Ethiopia, and DR Congo (range 3–188 g/day; figure 1). 17 (9%) of 185 countries, representing 1·8 billion people or 23·9% of the world's population, had mean consumption of at least one serving (100 g) per day.

**Table:** Estimated dietary consumption (g/day) of animal-source foods among children and adults, by world region, in 2018

	**World**	Southeast and East Asia*	**Central or Eastern Europe and Central Asia**†	**High-income countries**	**Latin America and Caribbean**	**Middle East and North Africa**	**South Asia**	**Sub-Saharan Africa**
**Unprocessed red meat**								
All ages	51 (48–54)	87 (79–96)	114 (101–126)	45 (43–47)	68 (64–72)	36 (31–43)	7 (7–8)	24 (23–26)
Children	40 (38–43)	84 (76–93)	93 (81–103)	30 (29–31)	65 (61–70)	36 (30–43)	7 (7–8)	20 (19–22)
Adults	56 (53–60)	88 (80–98)	121 (106–134)	50 (48–52)	70 (65–74)	37 (32–44)	7 (7–8)	29 (27–32)
**Processed meat**								
All ages	17 (15–21)	13 (10–17)	54 (45–64)	30 (28–32)	37 (32–43)	19 (11–31)	3 (1–12)	12 (6–26)
Children	18 (15–23)	26 (20–34)	44 (35–56)	25 (24–27)	37 (31–44)	18 (10–31)	3 (1–13)	12 (5–27)
Adults	17 (15–20)	8 (6–12)	57 (48–67)	31 (29–34)	37 (32–44)	19 (11–32)	3 (1–12)	12 (6–25)
**Seafood**								
All ages	28 (27–30)	44 (40–48)	30 (26–35)	25 (23–27)	22 (20–24)	23 (19–27)	12 (11–13)	31 (28–34)
Children	21 (20–23)	32 (30–35)	17 (14–20)	11 (11–12)	17 (15–19)	17 (14–21)	10 (9–11)	30 (27–33)
Adults	32 (30–34)	48 (44–53)	35 (30–41)	29 (27–32)	24 (22–27)	26 (22–32)	13 (12–14)	32 (29–36)
**Eggs**								
All ages	21 (18–24)	35 (27–45)	34 (28–42)	18 (16–22)	25 (23–27)	30 (26–35)	6 (5–7)	6 (5–7)
Children	17 (15–20)	34 (27–43)	30 (24–37)	14 (11–17)	25 (23–27)	29 (25–34)	6 (5–7)	5 (4–5)
Adults	23 (20–27)	35 (28–46)	35 (29–44)	20 (17–24)	25 (23–27)	30 (26–36)	6 (5–7)	8 (6–10)
**Milk**								
All ages	88 (84–93)	45 (41–51)	145 (129–165)	185 (173–201)	150 (140–161)	106 (87–131)	84 (77–90)	45 (41–49)
Children	103 (98–109)	75 (67–84)	166 (145–193)	252 (233–275)	185 (170–201)	127 (104–158)	94 (85–103)	46 (43–50)
Adults	81 (77–84)	35 (31–39)	138 (122–157)	165 (154–180)	133 (122–145)	93 (76–117)	78 (84–85)	44 (39–49)
**Cheese**								
All ages	8 (8–10)	2 (1–3)	34 (24–47)	32 (28–36)	13 (11–15)	17 (13–23)	1 (0–1)	1 (1–2)
Children	6 (6–7)	2 (1–5)	29 (20–42)	28 (25–32)	13 (11–16)	14 (11–19)	1 (0–1)	1 (1–1)
Adults	9 (8–11)	1 (1–3)	36 (25–50)	33 (29–37)	12 (10–14)	19 (15–25)	1 (0–1)	1 (1–2)
**Yoghurt**								
All ages	20 (17–23)	10 (7–14)	84 (59–125)	37 (32–46)	18 (15–20)	60 (46–80)	7 (6–8)	7 (5–10)
Children	18 (15–21)	11 (8–18)	73 (50–110)	37 (31–45)	23 (20–27)	52 (40–70)	7 (6–9)	6 (5–8)
Adults	21 (18–25)	9 (6–13)	87 (60–132)	37 (32–46)	15 (13–18)	64 (50–87)	7 (5–8)	8 (5–13)

Data are mean intake (95% uncertainty interval) in g/day. All intakes are reported adjusted to 700 kcal/day for ages <1 year, 1000 kcal/day for ages 1–<2 years, 1300 kcal/day for ages 2–5 years, 1700 kcal/day for ages 6–10 years, 2000 kcal/day for ages 11–74 years, and 1700 kcal/day for ages ≥75 years. Children were defined as individuals younger than 20 years and adults were defined as individuals who were aged 20 years or older. Data are based on a Bayesian hierarchical model that incorporated up to 499 individual-level dietary surveys per dietary factors, and additional survey-level and country-level covariates, to estimate dietary consumption levels. Standardised serving size used for this analysis: unprocessed red meat=100 g; processed meat=50 g; seafood=100 g; egg=55 g; cheese=42 g; yoghurt=245 g; milk=245 g.

* Referred to as Asia in previous Global Dietary Database reports.

† Referred to as the Former Soviet Union in previous Global Dietary Database reports.

Worldwide, mean intake of processed meat was 17 g/day (95% UI 15–21), with a 17-fold variation from highest (54 g/day; Central or Eastern Europe and Central Asia) to lowest (3 g/day; South Asia) regional intakes (table). Among the most populous countries, intakes ranged from 0 g/day to 62 g/day; highest in Germany, Russia, Philippines, and Brazil; and lowest in Bangladesh, India, Tanzania, and Turkey (figure 1). 32 (17%) of 185 countries, representing 520 million people or 6·9% of the world population, had mean intakes of at least one serving (50 g) daily.

Unprocessed red meat and processed meat intake were only moderately intercorrelated across countries (appendix p 28). Notable countries with much higher consumption of unprocessed red meat than processed meat included Russia (188 g/day *vs* 52 g/day), South Africa (147 g/day *vs* 17 g/day), and China (111 g/day *vs* 5 g/day; figure 1). In other countries, unprocessed red meat intake was lower than processed meat intake—eg, in the Philippines (25 g/day *vs* 45 g/day), DR Congo (12 g/day *vs* 30 g/day), Ethiopia (11 g/day *vs* 25 g/day), and Indonesia (26 g/day *vs* 37 g/day).

**Figure 2:**
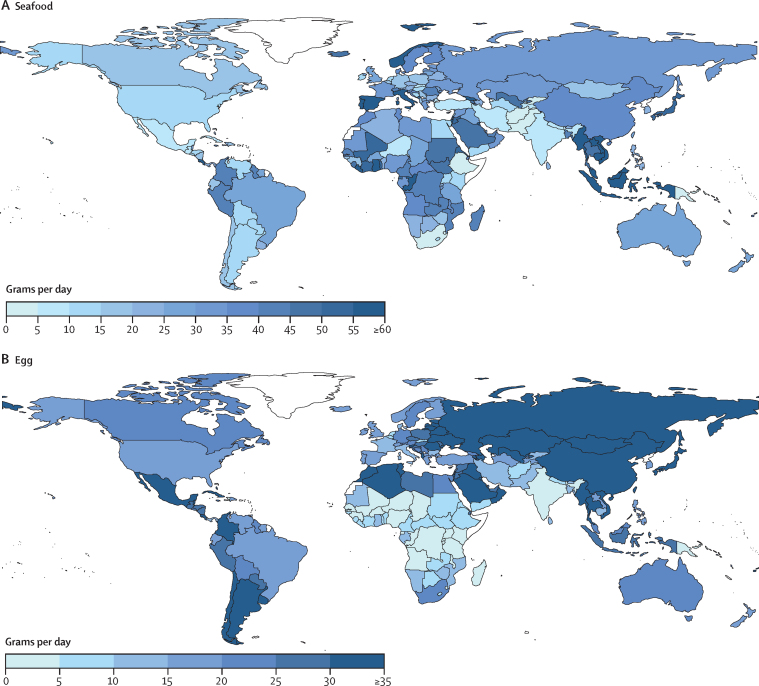
National mean intake of seafood (A) and egg (B) in 2018, all ages Only three (2%) of 185 countries consumed at least 100 g/day (equivalent to one serving per day) of seafood, representing 6·2 million people or 0·8% of the world population; five (3%) of 185 countries had intakes of at least 55 g/day (equivalent to one serving per day) of egg, representing 6·2 million people or 0·7% of the world population.

Mean global intake of seafood was 28 g/day (95% UI 27–30; table), with a 4-fold difference across regions (from 12 g/day in South Asia to 44 g/day in Southeast and East Asia). Generally, countries in Asia–Pacific and the Mediterranean basin had highest seafood intakes. Among the 25 most populous countries, mean national intakes were highest in Italy, Vietnam, Indonesia, and Japan (50–61 g/day), and lowest intakes were in Pakistan, Ethiopia, South Africa, and Turkey (4–6 g/day; figure 2). 87 (47%) of 185 countries had mean intakes of at least two servings (100 g each) per week, representing 4·0 billion people or 52·8% of the world population; and 16 of these countries had mean intakes of at least four servings per week (representing 37·5 million people, 5·0% of the world population). National seafood intake was not strongly correlated with unprocessed red meat or processed meat (appendix p 28).

Globally, mean consumption of eggs was 21 g/day (95% UI 18–24; table). Regional consumption was highest in Southeast and East Asia (35 g/day) and Central or Eastern Europe and Central Asia (34 g/day), and lowest in South Asia and Sub-Saharan Africa (both 6 g/day). Among the most populous countries, mean national intakes varied substantially from 1 g/day to 45 g/day; with lowest intakes in DR Congo, Tanzania, India, and Nigeria; and highest intakes in Vietnam, Japan, Russia, and China (figure 2). Only five (3%) of 185 countries consumed one or more servings of eggs (55 g) daily, representing 5·3 million people or 0·7% of the world's population. National egg intake was moderately correlated with unprocessed red meat and processed meat, but not with seafood (appendix p 28).

**Figure 3:**
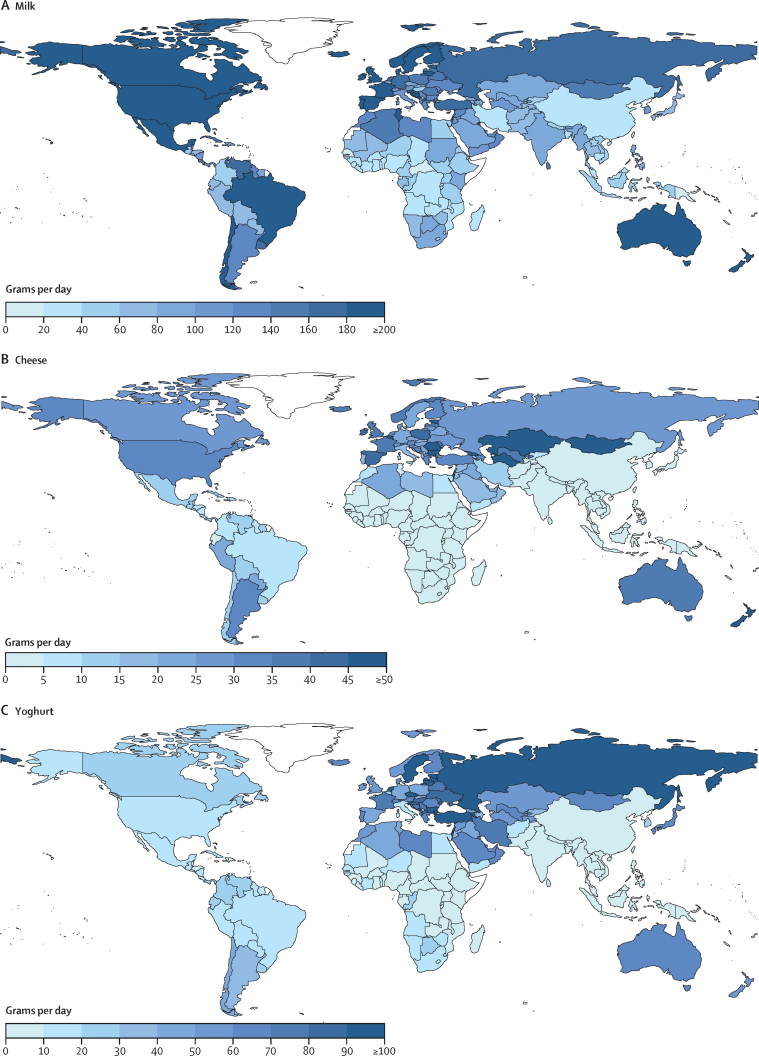
National mean intake of milk (A), cheese (B), and yoghurt (C) in 2018, all ages Only seven (4%) of 185 countries had intakes of at least 245 g/day (equivalent to one serving per day) of milk, representing 18·8 million people or 0·3% of the world population. 13 (7%) of 185 countries had intakes of at least 42 g/day (equivalent to one serving per day) of cheese, representing 173 million people or 2·3% of the world population. One (1%) of 185 countries had intakes of at least 245 g/day (equivalent to one serving per day) of yoghurt, representing 7 million people or 0·09% of the world population; 26 (14%) of 185 countries had intakes of at least 70 g/day (equivalent to 2 servings/week) of yogurt, representing 539 million people or 7·1% of the world population.

Worldwide, mean milk consumption was 88 g/day (95% UI 84–93), with 4-fold variation across regions (from 45 g/day to 185 g/day; table). Among populous countries, mean intakes were highest in Mexico, the UK, the USA, and France (188–206 g/day), and lowest in Nigeria, China, Bangladesh, and DR Congo (31–37 g/day; figure 3).

Mean global consumption of cheese was 8 g/day (8–10), with a 34-fold regional variation from 1 g/day in South Asia and sub-Saharan Africa to 34 g/day in Central or Eastern Europe and Central Asia (table). Among populous countries, highest intakes were in the UK, France, Turkey, and the USA (30–39 g/day; figure 3); and lowest intakes in DR Congo, Bangladesh, India, and Tanzania (≤1 g/day).

Mean global yoghurt intake was 20 g/day (95% UI 17–23; table), with regional consumption being at least 3-fold higher in Central or Eastern Europe and Central Asia (84 g/day) and the Middle East and North Africa (60 g/day), and only a third as much as the global mean in South Asia and sub-Saharan Africa (7 g/day each). National intakes ranged substantially, from 0 g/day to 503 g/day. Among populous countries, national intakes were lowest in Indonesia, Bangladesh, Tanzania, and Thailand (2–6 g/day) and highest in Turkey, Russia, Iran, and France (77–112 g/day; figure 3).

Compared with meats, national consumption of cheese, yoghurt, and milk were much more strongly intercorrelated (appendix p 28). National yoghurt intake was greater than milk intake only in Iran (71 g/day *vs* 38 g/day); and cheese intake was greater than yoghurt intake in the USA (30 g/day *vs* 14 g/day; figure 3).

**Figure 4:**
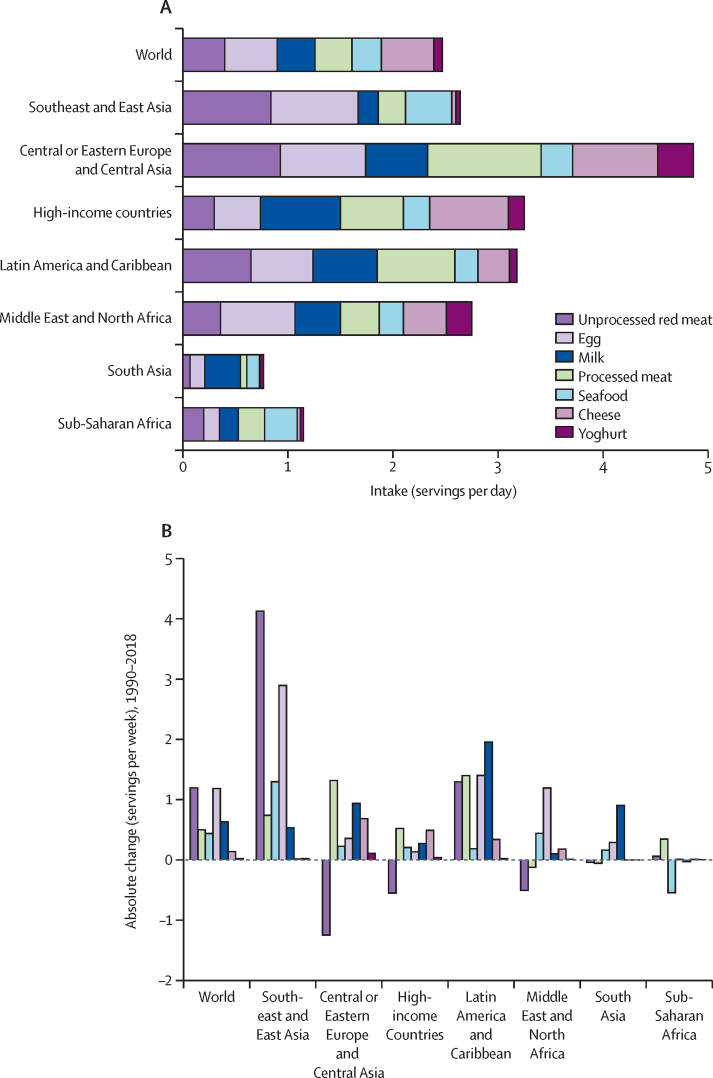
Mean global and regional consumption of animal-source foods (A) in 2018 and change in consumption between 1990 and 2018 (B) Positive values in part (B) indicate greater consumption in 2018 than 1900. One serving of unprocessed red meat=100 g; total processed meat=50 g; seafood=100 g; egg=55 g; cheese=42 g; yoghurt=245 g; milk=245 g. Uncertainty intervals for the absolute change in consumption between 1990 and 2018 are provided in the appendix (p 90).

Total consumption of ASF was lowest in South Asia (<1 serving per day) and sub-Saharan Africa (~1 serving per day), intermediate in Southeast and East Asia and the Middle East and North Africa (2–3 servings per day), higher in Latin America and Caribbean and high-income countries (3–4 servings per day), and highest in Central or Eastern Europe and Central Asia (~5 servings per day; figure 4). Among the most populous countries, total ASF consumption was highest in Russia (5·8 servings per day), Germany (3·8 servings per day), and the UK (3·7 servings per day), and lowest in India (0·7 servings per day) and Tanzania (0·9 servings per day; appendix p 37).

Globally and regionally, the mean energy-adjusted intakes of most ASF were not appreciably different between women and men (appendix pp 30–36, 61). Exceptions globally were yoghurt and milk, with women consuming slightly more yoghurt and milk than men (yoghurt 0·09 servings per week [95% UI 0·05 to 0·14; milk: 0·11 servings per week [0·02 to 0·20]) and slightly less processed meat (–0·31 servings per week [–0·49 to –0·14]).

Globally, intakes of most ASF increased with age, but age trends varied considerably at regional and national levels (appendix pp 38–53). Adults globally consumed more unprocessed red meat, seafood, and cheese than did children (unprocessed red meat: 56 g/day [95% UI 53–60] in adults *vs* 40 g/day [38–43] in children; seafood: 32 g/day [30–34] *vs* 21 g/day [20–23]; cheese: 9 g/day [8–11] *vs* 6 g/day [6–7]), whereas children consumed more milk (81 g/day [77–84] *vs* 103 g/day [98–109]; table). Larger regional differences in intake between adults versus children were found for some foods, such as for unprocessed red meat in Central or Eastern Europe and Central Asia, sub-Saharan Africa, and high-income countries; for processed meat in high-income countries, and Southeast and East Asia; for seafood in all regions except sub-Saharan Africa; and for milk in Southeast and East Asia and high-income countries.

**Figure 5:**
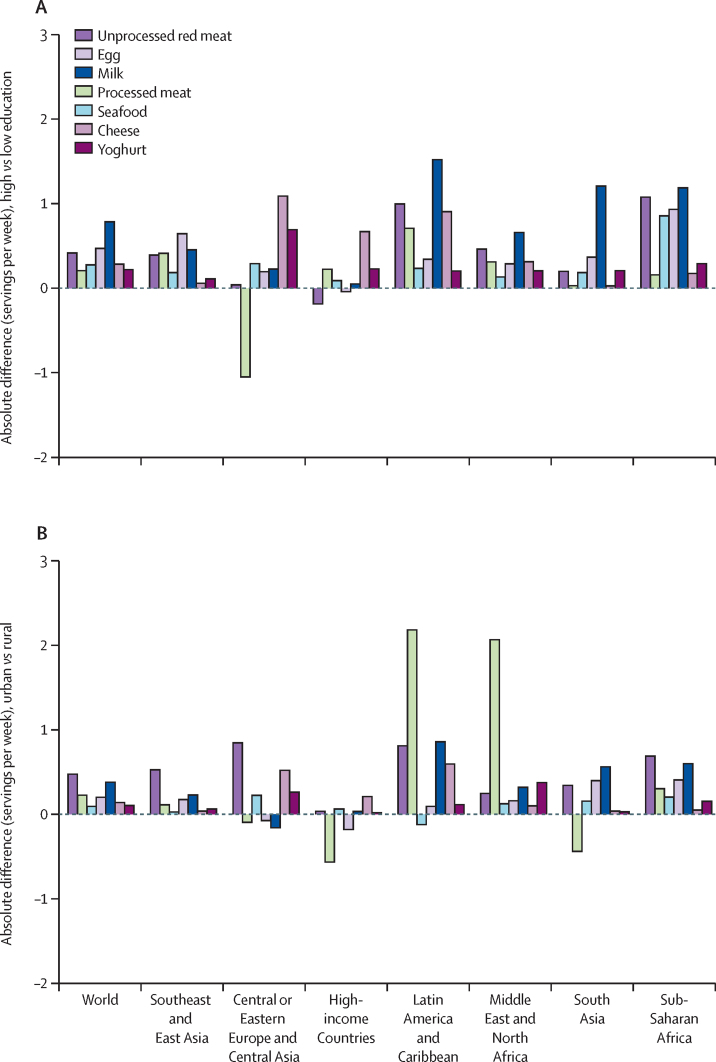
Mean global and regional absolute animal-source food intake difference by education level (A) and place of residence in 2018 (B) Positive values in part (A) indicate greater consumption in high-education level individuals, and in part (B) indicate greater consumption in individuals in urban areas. One serving of unprocessed red meat=100 g; total processed meat=50 g; seafood=100 g; egg=55 g; cheese=42 g; yoghurt=245 g; milk=245 g. Uncertainty intervals for the absolute change in consumption between 1990 and 2018 are provided in the appendix (p 81). Absolute difference by education level was computed as the difference at the stratum-level and aggregated to the global and regional mean differences using weighted population proportions for low (<6 years) and high education levels (≥12 years) only (excludes education level between 6 years and <12 years).

On average, individuals with higher education (≥12 years) *vs* low education (<6 years) consumed more ASF globally (figure 5, appendix pp 30–36, 70–78). In absolute servings, global differences by education were largest for milk (0·79 servings per week [95% UI 0·71–0·87]; 53·0% relative difference), followed by eggs (0·47 servings per week [0·36–0·60]; 50·6%), unprocessed red meat (0·42 servings per week [0·35–0·49]; 29·1%), cheese (0·28 servings per week [0·23–0·34]; 78·7%), seafood (0·28 servings per week [0·23–0·33]; 19·8%), yoghurt (0·22 servings per week [0·18–0·27]; 135·8%), and processed meat (0·21 servings per week [0·08–0·35]; 73·7%). In all regions, more educated individuals consumed more milk, except in Central or Eastern Europe and Central Asia and high-income countries where intake by education was more similar. The largest regional difference in absolute servings by higher education was seen for unprocessed red meat, seafood, and eggs in sub-Saharan Africa, and processed meat, cheese, and yoghurt in Central or Eastern Europe and Central Asia.

Compared with rural individuals, mean global intakes were higher among urban individuals for all ASF except processed meat (figure 5; appendix pp 30–36, 79–87). The largest global differences (in absolute intakes) were for unprocessed red meat (0·47 servings per week [95% UI 0·40–0·55]; 35·7% relative difference), milk (0·38 servings per week [0·30–0·46]; 23·9%), and eggs (0·20 servings per week [0·06–0·34]; 31·1%). The largest regional difference in absolute servings by urban residence was seen for processed meat, milk, and cheese in Latin America and Caribbean, unprocessed red meat in Central or Eastern Europe and Central Asia, seafood and eggs in sub-Saharan Africa, and yoghurt in the Middle East and North Africa.

Between 1990 and 2018, mean unprocessed red meat intake per person increased globally by 88·1%, equivalent to an additional 1·20 servings per week (95% UI 1·06 to 1·35; standardised to 2018 population distributions; figure 4; appendix pp 88–96). Notably, this global trend was due to increased intake in only three of the seven regions: Southeast and East Asia increased by 4·12 servings per week (3·66 to 4·64; 265·7% relative difference); Latin America and Caribbean increased by 1·29 servings per week (1·19 to 1·41; 57·9%); and sub-Saharan Africa increased by 0·06 servings per week (0·05 to 0·07; 26·3%). Intake decreased in Central or Eastern Europe and Central Asia, Middle East and North Africa, high-income countries, and South Asia. Among populous countries, absolute increases were largest in China (increase of 5·89 servings per week [5·18 to 6·69]; 312·5%), Japan (3·53 servings per week [3·36 to 3·71]; 128·8%), Brazil (2·45 servings per week [2·22 to 2·72]; 94·3%), and Mexico (1·18 servings per week [1·08 to 1·28]; 55·7%). Absolute decreases were largest in Russia (–2·00 servings per week [–2·34 to –1·30]; –14·0%), Germany (–1·19 servings per week [–1·33 to –1·07]; –26·3%), Iran (–1·04 servings per week [–1·16 to –0·92]; –47·7%), and France (–0·96 servings per week [–1·08 to –0·84]; –22·1%).

Between 1990 and 2018, mean processed meat intake increased globally by 152·8% (0·50 servings per week [95% UI 0·27 to 0·71]), with increases in most regions (0·35–1·40 servings per week) except in the Middle East and North Africa and South Asia where intakes were stable (figure 4; appendix pp 88–96). Among populous countries, the largest increases were in the Philippines (3·94 servings per week [3·13 to 4·90]; 163·2%), Brazil (3·82 servings per week [2·82–4·91]; 186·7%), Indonesia (2·57 servings per week [1·15–4·36]; 410·4%), and Russia (2·54 servings per week [1·54–3·76]; 53·9%). Only two (8%) of 25 populous countries had decreases: Nigeria (–0·75 servings per week [–2·81 to –0·02]; –33·0%) and Mexico (–0·71 servings per week [–1·02 to –0·42]; –21·6%).

Global seafood consumption doubled (109·4%) between 1990 and 2018, increasing by 0·44 servings per week (95% UI 0·37–0·51; figure 4; appendix pp 88–96). Increases were seen in all regions except sub-Saharan Africa, with the largest absolute increase in Southeast and East Asia (1·30 servings per week [1·10 to 1·51]; 148·7%). Among populous countries, the largest increases occurred in Vietnam (3·22 servings per week [2·23 to 4·69]; 306·1%), Thailand (1·68 servings per week [1·17 to 2·42]; 176·2%), China (1·66 servings per week [1·41 to 1·94]; 167·3%), and Italy (1·38 servings per week [1·22 to 1·56]; 47·4%). The largest absolute decreases were in Tanzania (–8·01 servings per week [–9·62 to –6·55]; –81·1%), the Philippines (–3·22 servings per week [–3·53 to –2·94]; –65·2%), and Japan (–2·21 servings per week [–2·42 to –2·01]; –38·6%).

Globally, egg consumption per person increased by 141·4% between 1990 and 2018, rising by 1·18 servings per week (95% UI 0·94 to 1·50). Regional intakes increased by 2·89 servings per week in Southeast and East Asia (2·14 to 3·91; 345·5%) to 0·29 servings per week in South Asia (0·24 to 0·34; 54·1%; figure 4, appendix pp 88–96). Across populous countries, the greatest increases were in Vietnam (4·90 servings per week [3·63 to 6·56]; 564·3%), China (3·67 servings per week [2·62 to 5·10]; 410·2%), Mexico (2·57 servings per week [2·36 to 2·80]; 123·6%), and South Africa (1·28 servings per week [0·92 to 1·74]; 112·9%). Ethiopia had the largest decrease in egg intake (–0·58 servings per week [–0·70 to –0·48]; –41·0%), followed by Germany (–0·48 servings per week [–0·53 to –0·43]; –13·8%), Tanzania (–0·46 servings per week [–0·56 to –0·37]; –48·0%), and France (–0·18 servings per week [–0·20 to –0·15]; –11·0%).

Changes over time in intakes of milk, cheese, and yoghurt are summarised in the appendix (pp 29, 88–96). Milk consumption doubled globally, cheese consumption increased by 56·0%, and yoghurt consumption did not change significantly (figure 4).

## Discussion

Our systematic analysis, based on GDD data evaluating 499 largely national, individual-level dietary surveys, provides new estimates of global, regional, and national consumption of ASF between 1990 and 2018. Several aspects of these findings are novel, including the results for children and the overall population stratified by education level and by urban or rural residence. These are also, to our knowledge, the first worldwide estimates for intakes of cheese and yoghurt. The overall findings have important implications for both human and planetary health.

Worldwide, unprocessed red meat intake per person increased by 88·1% over this period (increases would be larger further accounting for population growth), but almost entirely due to increases in Southeast and East Asia and Latin America and Caribbean; modest decreases were found for most other regions. Limiting intake of unprocessed red meat is nutritionally recommended in many countries through national food-based dietary guidelines due to links with cardiovascular disease, diabetes, and certain cancers.^14^, ^15^ At the same time, livestock production using current technologies is the single greatest contributor to greenhouse gas emissions from the agriculture sector (5·8% of global greenhouse gas in 2016).^42^ Our results suggest that greater unprocessed red meat intakes over time in particular countries with high populations—especially China, Japan, Brazil, and Mexico—run counter to these recommendations for moderation. Consistent with our findings, the China National Nutrition Surveys/China Health and Nutrition Survey and the FAO Food Balance Sheet data show that red meat consumption and availability substantially increased over time, largely due to increased pork consumption.^35^, ^43^ Our findings also show higher intakes among more educated and urban individuals, suggesting that continuing global advances in education and urbanicity might further exacerbate global trends.

Intake of processed meat also increased, by 152·8% globally, with increases in most world regions. Notably, intakes of unprocessed red meat and processed meat were only moderately correlated across countries, and processed meat intake was not associated with urbanicity globally. These findings suggest differential drivers—and potential levers for action—for unprocessed versus processed meats, with urbanisation having less influence on processed meat consumption compared with socioeconomic factors.^44^, ^45^ This factor is an important consideration for influencing total meat consumption globally, given the generally stronger links with NCDs of processed meats.

Seafood has been shown to be an important component of a healthy diet, including for childhood brain development^46^, ^47^ and cardiovascular health in adults.^48^, ^49^ Global seafood consumption doubled after 1990, and by 2018 more than half of countries had mean intakes of two or more servings per week. However, seafood intake remained low in many South Asian, Latin American and Caribbean, and Middle Eastern and North African countries, with only small improvements over time. Adjusted for energy, seafood intake was also generally much lower among children than adults in most world regions, and among those with lower education or rural residence. These new results suggest important disparities that must be addressed, together with sustainable approaches to increase seafood production,^16^ to ensure adequate health benefits for all.

Global dietary intakes also increased for eggs (by 141·4%), milk (98·6%), and cheese (56·0%), whereas yoghurt intake was stable. However, absolute intakes remained relatively low in most countries, with few countries reaching intakes of one daily serving for each. National intercorrelations of eggs and dairy foods were relatively high, and generally consistent with population prevalence of lactase deficiency.^50^ Consistent with high levels of lactase deficiency, some countries in Asia had high intakes of eggs only. Conversely, most sub-Saharan African countries had low intakes of eggs and dairy, with very little change between 1990 and 2018. These findings highlight the importance of strategies to augment intakes of these lower cost, more environmentally sustainable animal food sources of nutrients,^16^, ^17^ while also accounting for genetically driven challenges in tolerance for dairy.

The Eat–*Lancet* Commission on Food, Planet, and Health's report proposed common targets for a healthy and environmentally sustainable diet, including limiting red meat, poultry, and egg consumption, and moderate levels of fish and dairy consumption.^16^ The impact of several of these targets for health versus sustainability may be quite different. For example, reducing unprocessed red meat intake to the suggested targets would have large impacts on sustainability but much smaller effects on health.^4^ Reducing dairy, poultry, and eggs to the targets would have smaller sustainability impacts^4^ and little health benefit,^51^ and some populations may benefit from increasing their currently low intakes of these foods closer to the targets.^29^, ^52^, ^53^ Our findings show that few countries in 2018 met the Eat–*Lancet* target for reduced total red meat (3·8% of countries, based on a target of ≤98 g/week of unprocessed red meat and processed meat combined [processed meat included processed poultry]), less than half for fish (43·2% of countries ≤196 g/week of seafood), and eggs (33·5% of countries ≤91 g/week), but most for dairy (83·8% of countries ≤250 g/day of milk, cheese, and yoghurt combined).

Previous studies of global ASF consumption include fewer current data, evaluated fewer ASF subtypes, and did not include children.^21^, ^22^, ^23^ Consistent with our findings, the Global Burden of Diseases, Injuries, and Risk Factors Study (GBD) study estimated that milk intake was highest in Australasia, western Europe, and North America, and lowest in South Asia and sub-Saharan Africa.^23^ Our global mean estimates for unprocessed red meat and processed meat consumption were higher than the GBD study's estimates, although both studies generally found high meat consumption in North America, western Europe, and Latin America, and low consumption in south Asia and sub-Saharan Africa. Differences in estimated intakes and trends might be attributable to the larger number of individual-level dietary surveys in the GDD, reliance on national food availability data as estimates of individual-level dietary data in the GBD, and differing modelling methods.

Our study has several strengths. Global ASF intakes were estimated using 499 dietary surveys, mostly nationally representative and assessing individual-level dietary intakes and including 3·8 million individuals from 134 countries representing 95·1% of the world's population. Data were harmonised using standardised protocols, with Bayesian hierarchical modelling to incorporate survey and country covariates and address heterogeneity, missingness, and sampling and modelling uncertainty. Findings were assessed over time and by important demographic characteristics including age, sex, education, and urban or rural residence.

Potential limitations warrant mention. Survey availability was limited for some (particularly low-income) countries, demographic groups, time periods and ASF subtype (eg, <200 surveys on cheese and yoghurt *vs* >400 on milk and unprocessed red meat), increasing uncertainty in these estimates. All types of dietary data are measured with some error, including from individual-level surveys as well as national food availability estimates. Additionally, the standardisation of available global data required certain decisions and assumptions about serving sizes, food group definitions, energy adjustment, and the disaggregation of household-level data when standardising the dietary surveys. However, we utilised rigorous methods developed over 14 years of work and carefully documented and detailed each survey's methods and standardisation process to maximise transparency in our methods. This iteration of the GDD did not collect information on poultry, an important ASF in many countries globally. Last, our analysis was limited to the consumption of ASF and does not describe trends in ASF production. Overall, these new results should be considered the best currently available, rather than perfect estimates of dietary intakes of ASF worldwide. In addition, our findings identify specific world regions and countries most urgently requiring well-conducted national surveys on individual-level intakes of ASF.

## Data sharing

The modelled estimates of animal-source food intakes by population subgroup, country, region, and globe in 1990 and 2018 are available for download from the Global Dietary Database. Survey-level information and original data download weblinks are also provided for all public surveys; and survey-level microdata or stratum-level aggregate data are provided for direct download for all non-public surveys granted consent for public sharing by the data owner.

## Declaration of interests
