# Correction to *Lancet Planet Health* 2022; 6: e243–56

**DOI:** 10.1016/S2542-5196(24)00233-X

**Published:** 2024-09-10

*Miller V, Reedy J, Cudhea F, et al. Global, regional, and national consumption of animal-source foods between 1990 and 2018: findings from the Global Dietary Database.* Lancet Planetary Health 2022; **6:** e243–56—In figure 4, panel A of this Article, some of the values for unprocessed red meat consumption were slightly smaller and some values for egg and cheese consumption were slightly larger than the correct values given in table 1. The figure has been corrected on Sept 10, 2024.

